# Psychological Mobilization of Innovative Teaching Methods for Students' Basic Educational Curriculum Reform Under Deep Learning

**DOI:** 10.3389/fpsyg.2022.843493

**Published:** 2022-06-09

**Authors:** Dingzhou Zhao, Hongming Li, Annan Xu, Tingchang Song

**Affiliations:** ^1^Physical Education College of Zhengzhou University, Zhengzhou, China; ^2^Southampton Education School, University of Southampton, Southampton, United Kingdom; ^3^School of Mathematics and Statistics, Shaanxi Normal University, Xi'an, China; ^4^School of Management, Chongqing Institute of Engineering, Chongqing, China

**Keywords:** education innovation, deep learning, edge computing, neural network, psychological factors

## Abstract

Educational innovation reform is used as the background. In response to the need to propose innovative educational programs, the concepts of Distributed Deep Neural Network (DDNN) and deep learning under edge computing are used as the basis. A teaching program for Science Technology Engineering Mathematics (STEM) is proposed. The average training method is used to verify the performance of the model. Sampling rate means the number of samples per second taken from a continuous signal to form a discrete signal. The accuracy and sample ratio obtained are higher than 95%. The communication volume is 309 bytes, which is in a good range. On this basis, a university uses STEM teaching plans and questionnaires to influence the psychological mobilization factors of students' deep learning effects. Challenging learning tasks and learning motivation have the greatest impact on deep learning, and conclusions that both are positive effects are obtained. Therefore, STEM innovative teaching programs can be widely used. The plan provides a reference theory for improving teaching innovation in the context of the basic educational curriculum reform in China. STEM curriculum is the dual subject of teachers and students, and the learning community includes multi-stakeholders. There are hierarchical relationships among the subjects. In terms of financial support, the first two funds come from the school. Learning communities have dedicated sponsorship partners complemented by clear financial planning. There is not much difference in course resources. Still, the learning community will provide more diversified media forms and special websites, and other auxiliary resources are open to all users. They can obtain first-hand resources without applying. In terms of project form, in addition to the core classroom teaching, the latter two can provide richer activities and realize the diversity of time, space, and information exchange.

## Introduction

Since the implementation of China's education reform policy, teaching personnel in the education field have been optimized to different degrees in terms of their overall quality and establishment structure. The problem of incomplete training structure still exists. The structural construction of vocational education emphasizes the overall improvement of the quality of talents and the balance of technical talents (Schreuder and Noorman, 2019). In this era, technical talents must be comprehensively cultivated, and the skill level and quality level of professional teachers must be improved (Graham et al., 2020). Therefore, China's Ministry of Education (MOE) has proposed a plan to improve the quality of teachers. The plan requires teachers to improve the traditional teaching methods and use innovative teaching methods to improve technology to cultivate the psychological interest of middle school students in learning (Govindan and Regina, 2018). Especially in higher education, there is a serious imbalance between the cultivation of theoretical knowledge and the improvement of practical ability. This has led to the fact that the knowledge and technical mastery of technical talents are not as many as those of theoretical majors (Asngari and Sumaryant, 2019). Therefore, innovative teaching methods are proposed.

As the policy is put forward, many scholars and professional talents began to explore innovative education methods. Wu and Song (2019) explored the use and satisfaction of social media in entrepreneurship courses from the perspective of learners in order to understand the status quo of learners' use of social media in entrepreneurship courses. The result reveals that trust, profit, learning, and social interaction are the three elements to satisfy learning psychology. Especially the elements of trust are worthy of being studied in the study courses (Wu and Song, 2019). Innovative education must improve the psychological quality of students. Radoslaw et al. (2018) believed that fragile narcissism and highly competitive scores indicate fragile self-esteem. A high score of admiration indicates the best self-esteem. The competition is between fragile narcissism and admiration. This supports its positioning in the self-importance dimension of the narcissism spectrum model (Radoslaw et al., 2018). Wu et al. (2019) believed that narcissism or spiritual quality has a certain influence on the process of education, entrepreneurship and practice (Wu et al., 2019). In recent years, among the various innovative teaching methods that have been proposed, the deep learning model is a widely used method. It uses projects and challenges to help students gain more motivation for active learning in the classroom and ensure their learning motivation. Young et al. (2020) investigated a cognitive learning model for deep learning in the traditional classroom of educators. Innovative methods that deep learning can better provide teaching have been discovered (Young et al., 2020). Fernández and Fraga (2019) used edge computing and the Internet of Things (IoT) to drive applications in the IT facilities of a smart campus, and found that they can effectively provide useful guidance for developers (Fernández and Fraga, 2019). The above literature shows that many emerging technologies have been used in the field of innovative education. Therefore, among all the factors that affect students' deep learning, the factors of learning psychology are discussed. Science Technology Engineering Mathematics (STEM) education is not only a change in teaching content, nor a simple combination of various subjects, but a new educational practice. Integration and innovation are the directions of continuous breakthroughs in STEM education in the future. This not only needs to integrate the knowledge of each subject but also needs to deal with the relationship between each subject curriculum and the corresponding period of study. More importantly, it is urgent to develop a truly scientific spirit while learning technical knowledge to solve practical problems. Modern STEM education not only promotes skills such as critical thinking, problem-solving, higher-order thinking, design, and reasoning, but also behavioral competencies such as perseverance, adaptability, cooperation, organization, and responsibility.

With the advent of big data and efficient computing resources, deep learning has made major breakthroughs in many fields of artificial intelligence (AI). However, in the face of increasingly complex tasks, the scale of both data and deep learning models has become increasingly large. For example, the amount of labeled image data for training image classifiers are in the millions or even tens of millions. The emergence of large-scale training data provides a material basis for training large models. Therefore, in recent years, many large-scale machine learning models have emerged in society. However, when the training data vocabulary grows to tens of millions, deep learning models can have billions of parameters without any processing. In order to improve the training efficiency of deep learning models and reduce training time, distributed deep neural networks (DDNN) are commonly used to perform training tasks. Additionally, multiple worker nodes are used to train neural network models with good performance in a distributed and efficient manner. Distributed technology is an accelerator of deep learning technology, which can significantly improve the training efficiency of deep learning and further expand its application scope. In deep learning for students, the factors of learning psychology are used as research purposes. Edge computing is an important technology in IoT services. Deep learning combined with DDNN under edge computing technology, STEM teaching system is proposed. In terms of theory and practice, the mutual promotion between STEM teaching methods and deep learning is explored. The STEM teaching model is trained and the resulting data is viewed. The questionnaire method is adopted. During the learning process of innovative teaching, students in a certain school have been investigated about their learning motivation and learning effects. Through the data of the questionnaire, the feasibility of innovative teaching methods is analyzed. Edge computing and deep learning are combined, and the STEM teaching system is proposed. The system is applied in teaching work.

## Methods

### Deep Learning Concepts

**Figure 1:**
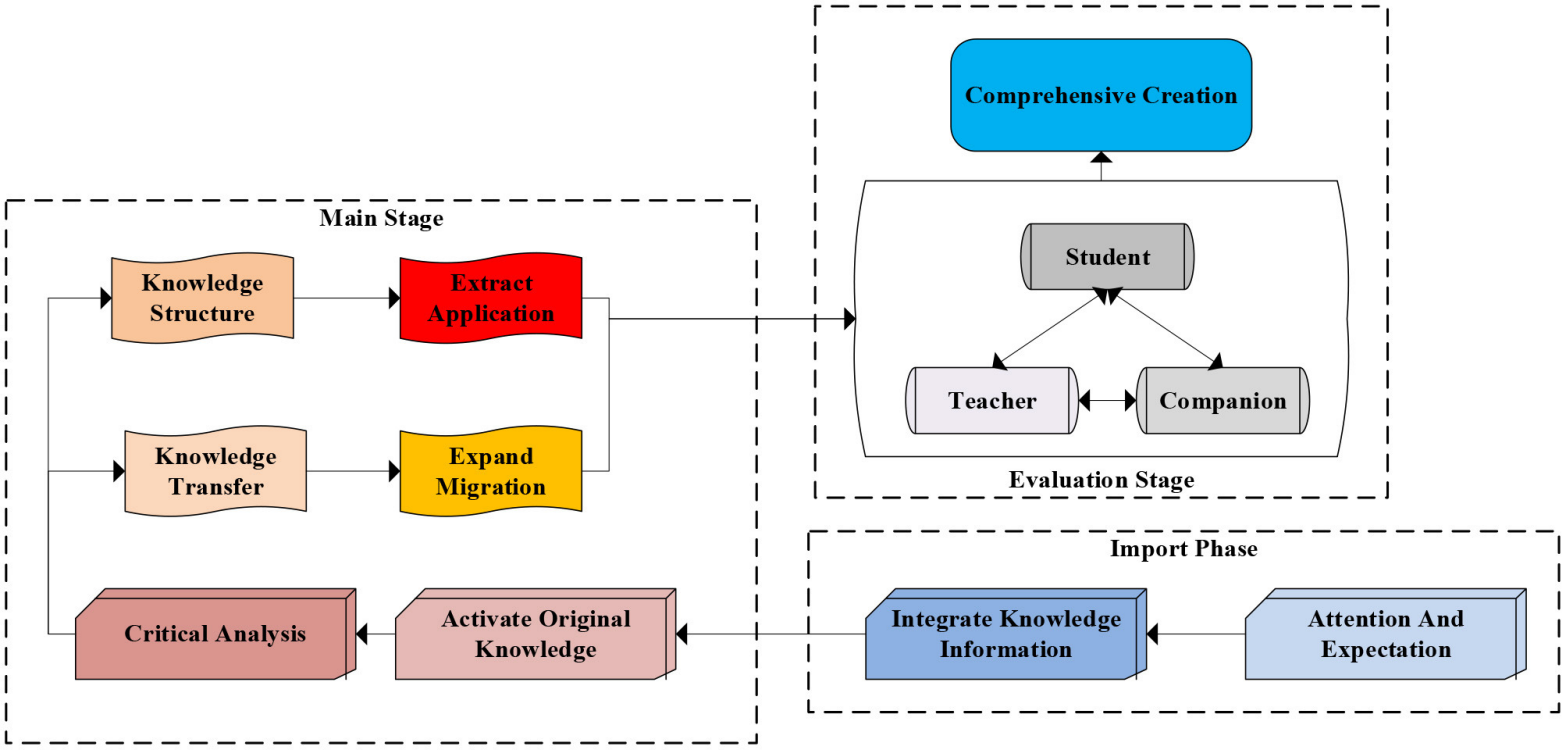
The process of deep learning.

The learning community reflects people's attention to the socialized learning form that goes beyond the teaching situation of the school class. This reflects the value pursuit of the social construction of knowledge. The ideological core of the learning community is the sociality of knowledge construction, cultural field, subject interaction, wisdom sharing, and environmental support. The National Research Council of the United States puts forward the STEM learning ecosystem view, which takes the learner as the center and regards various elements related to learning as a system, emphasizing the dynamic interaction between learners, diverse scenarios, learning communities, and cultural elements. Distributed deep learning neural network technology has an excellent performance in speech recognition, image handwriting recognition, and other fields. It enables machines to see images and texts with “eyes” and listen to sounds with “ears” like humans, thus possessing perceptual intelligence. Combining it with technologies such as reinforcement learning enables machines to reorganize and reason about knowledge, thereby possessing higher levels of cognitive intelligence. Therefore, AI technology based on deep learning will bring profound changes to the field of education. The essence of deep learning is a multi-level learning method that focuses on motivation, knowledge, ideas, state and thinking (Wataya et al., 2020). Its structure is shown in Figure 1. When guiding students in deep learning, teaching concepts should be constructed according to the core. As a new learning method, shallow learning is only for learners to obtain simple, low-level cognition (Dumford and Miller, 2018). While deep learning is to integrate information fragments and combine subject knowledge to build a bridge between new and old knowledge to promote understanding and application. Deep learning mainly focuses on several characteristics as shown in Table 1.

**Table 1:** Four elements and connotations of deep learning.

**Name**	**Meaning**
Meaningful learning	Meaningful learning covers five characteristics: 1. in the learning process, learners should be active; 2. knowledge acquisition is through construction rather than reproduction; 3. learning should be targeted; 4. the learning process should take place in a complex or real situation; 5. learners should cooperate with each other.
Knowledge system construction	The process of acquiring knowledge is not simply the learner's acceptance of knowledge points, but the learners encounter problems or tasks in life. Combine all their knowledge to compare and combine, find similar connections, and build their knowledge system. This series of processes should be their positive will.
Critical thinking	This is the ability to make rational judgments and objective summaries using sufficient and reasonable objective events. It is a reasonable and reflective thought.
Metacognitive ability	Metacognition is the re-cognition of the cognitive process. In short, it is the ability to properly adjust and recognize the degree of knowledge related to the entire cognitive process. It is an individual's self-discovery, judgment, evaluation, and adjustment of the knowledge processing process.

Many research experts and scholars have summarized the connotation of deep learning as follows: deep learning is a constructive learning process that learns actively and has a critical attitude in the mind. Four factors are covered in deep learning, meaningful learning, knowledge system construction, critical thinking, and metacognitive ability (Chen and Ran, 2019). When these factors co-occur and are promoted, the process of deep learning can be triggered. These four factors all belong to high-order thinking ability, which means that the factors emphasized by deep learning are consistent with the learning ability of high-order thinking. The connotation explanations of the four factors are shown in Table 1.

### The Concept and Construction Method of STEM Teaching Plan

**Figure 2:**
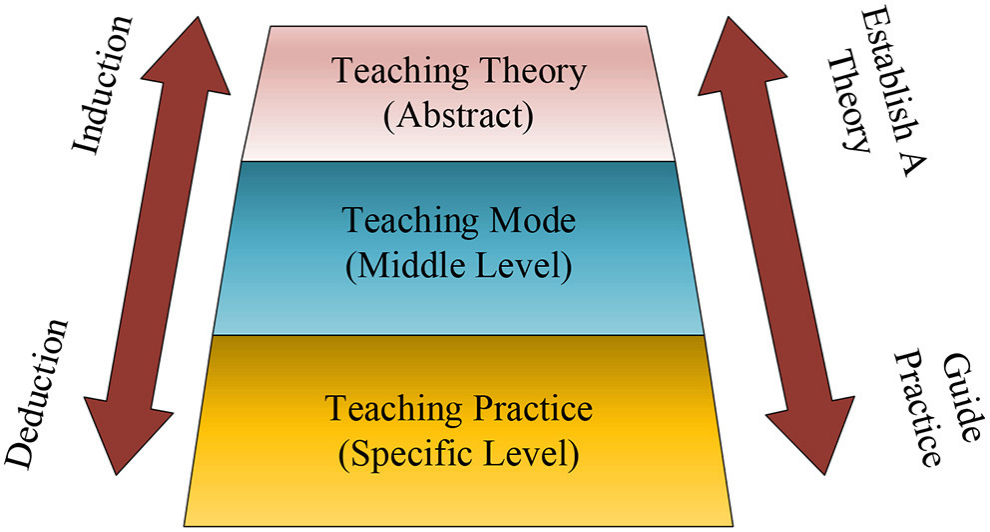
Structure model diagram of STEM teaching method.

STEM comes from the abbreviation of four disciplines. S stands for Science, T stands for Technology, E stands for Engineering, and M stands for Mathematics (Ugraş and Genç, 2018). This teaching philosophy originated in the United States. Talents with scientific and technological innovation capabilities and technological application capabilities cultivated through STEM teaching programs are key factors for the country's continued development and technological innovation. Under normal circumstances, to teach knowledge to students, a complete set of teaching process is divided into five factors: theoretical basis, teaching objectives, teaching measures, auxiliary conditions, and evaluation summary. Among them, STEM teaching method is one of innovative teaching methods. Compared with the traditional teacher-centered teaching methods in large classes, STEM teaching methods emphasize students as the main body. As the initiator and assistant in the teaching process, the teacher is responsible for creating suitable problem situations. While teaching, the teacher divides the students into groups for cooperation and exploration. The members of a group have the same goal, and cooperate with the knowledge they possess to acquire new knowledge and solve the problems raised by teachers, and master comprehensive skills. This teaching method has greatly mobilized students' learning motivation and enthusiasm. Stimulate creative thinking and ability (Mutakinati et al., 2018). The STEM teaching method serves as a bridge between the theoretical foundation of teaching and the comprehensive practice of teaching, and the structure is shown in Figure 2.

**Figure 3:**
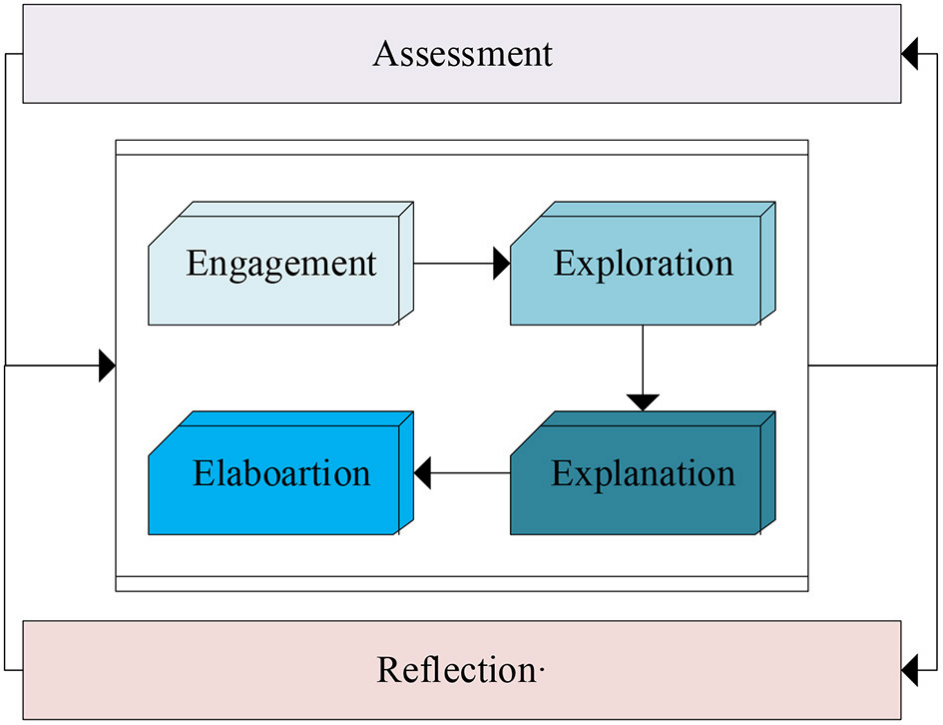
STEM teaching structure of 4Ex2.

Figure 3 shows the 4Ex2 teaching structure under STEM teaching mode (Alkhatib, 2018). Among them, 4E refers to the engagement, exploration, explanation and elaboartion. x2 is evaluation and summary reflection. This teaching method can help teachers design deeper topics to explore and ease, and emphasizes the evaluation of students in the entire process.

**Figure 4:**
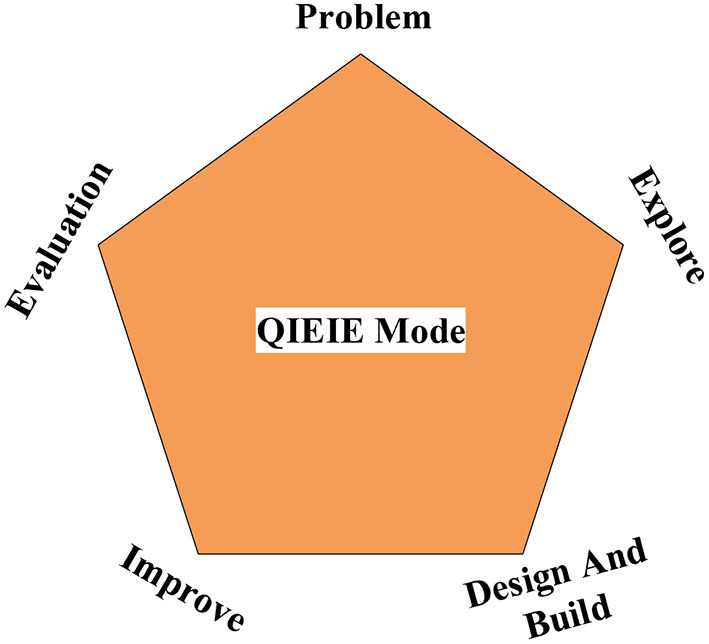
QIEIE teaching structure.

Figure 4 shows the QIEIE teaching model widely used in China (Camilli and Hira, 2019). This structure is designed using the specific curriculum background. Since 4Ex2, the design in engineering serves as a connection, emphasizing the knowledge of practical technology, science, and science. It connects the learning process of students. The process of Question, Investigation, Excogitation, Improvement, Evaluation combines the characteristics of various disciplines in China.

### STEM Teaching Mode Combined With DDNN Model in Edge Computing

At present, the widely used traditional machine learning methods have poor accuracy in using raw data for model calculation. On this basis, the improvement measure is to convert it into the data format with feature vector, and then send it to the learning machine for processing. Therefore, a Distributed Deep Neural Network (DDNN) model for edge computing is proposed. The structure of DDNN includes convolution layer, pooling layer, and fully connected layer (Lee et al., 2020). The convolution layer contains two training parameters: the weight of convolution kernel and deviation term. The operation process of convolution kernel is to perform the operation through the translational motion in the input data graph. For two-dimensional processes, there are three dimensions in the convolution kernel. The height, width, and number of channels are obtained by matching the number of convolution kernel channels with the number of data channels one by one to obtain the inner product. Then, sum the data and obtain the result of convolution. The feature extraction module of the underlying convolution layer of DDNN processes the data to obtain the data features with the underlying expression. After many convolution calculations and non-linear changes, it is transformed into high-level abstract features, which is more inclined to the semantic information of the target data. Therefore, DDNN is suitable for calculating complex functions.

A major bottleneck in scaling the distributed training process is the high bandwidth cost of model weights and gradient communication between nodes. This bottleneck is especially pronounced when training on devices using federated learning. It has the problem of low network bandwidth and slow connection speed. In response to this problem, scholars have proposed various methods for the efficient utilization of network bandwidth. It uses asynchronous and synchronous variants to allow nodes to communicate independently of each other but also enables parallelization and improves network bandwidth utilization to a certain degree. In addition, scholars have made some significant progress in gradient compression. These methods are mainly based on two main ideas: quantization and sparsification.

DDNN can be applied to edge computing and the cloud. There is a classifier on one side of each device, one on the edge and one on the cloud. They form a multi-outlet cascade classifier (Hassen et al., 2021). Among them, each outlet takes the loss of cross-entropy as the main optimization goal, as shown in equations (1), (2), and (3):


(1) $$\begin{array}{r} \text{L}\left(\text{y},\text{y};\text{θ}\right)\;=\;\frac{1}{\left|\text{C}\right|}\sum_{\text{c}\in \text{C}}{\text{y}}_{\text{c}}\log\left({\text{y}}_{\text{c}}\right) \end{array}$$

(2) $$\begin{array}{r} \text{y }=\text{ softmax}\left(\text{z}\right)\;=\;\frac{\text{exp}\left(\text{z}\right)}{\sum_{\text{c}\in \text{C}}\text{exp}\left({\text{z}}_{\text{c}}\right)} \end{array}$$

(3) $$\begin{array}{r} \text{z }=\;{\text{f}}_{\text{exi}{\text{t}}_{\text{n}}}\left(\text{x};\text{θ}\right) \end{array}$$

*L*(*y, y*; θ) is the loss that will export the cross entropy. x is the data sample of the data neural network structure, y is the real label of these data samples, C is the set of these real labels, *f*_*exi*_*t*__*n*__ is the operation of the data sample from the input of the neural network to the nth exit process, θ is the weight value and bias value of the neural network in this process.

Equation (4) is the loss function of DDNN. The loss weights for each exit (Rengasamy et al., 2020) are summed and trained. The gradient descent method is used to update the parameters in the DDNN. After the training process is over, the classification of each exit is compared for accuracy. Compared with depth, its accuracy has reached a balanced state.


(4) $$\begin{array}{r} \text{L}\left(\text{y},\text{y};\theta \right)\sum_{\text{n }=\;1}^{\text{N}}\omega_{\text{n}}L\left({\hat{\text{f}}}_{{\text{exit}}_{\text{n}}},y;\theta \right) \end{array}$$

N is the total number of classifiers exits, ω_*n*_ is the weight of each classifier export, and ${\hat{f}}_{exit_{n}}$ is the estimated number of exits of the n-th classifier. The training process of DDNN is carried out through cloud services (Torres et al., 2021).

Equation (5) calculates the confidence of the end-side exit, as shown in the equation (5):


(5) $$\begin{array}{r} \eta \left(x\right)\;=\;-\sum_{\text{i }=\;1}^{\left|\text{C}\right|}\frac{{\text{x}}_{\text{i}}\log x_{\text{i}}}{\log|C|} \end{array}$$

The reasoning process of DDNN is closely related to the number of exits of the classifier. In the case of only one outlet, the data of the sensors connected to the device must all be sent to the cloud for calculation. When there are exits on the end side, edge side, and cloud side, a threshold is set at each exit. The confidence level calculated using equation (5) is compared with the manually set threshold. If the threshold is lower than the confidence level, the feature vector on the end side is sent to the server on the edge side for calculation. The calculated confidence on the edge side is compared with the threshold. If the classification result meets the experimental requirements, the data will be output from the side exit of the calculation.

Another important component of distributed training is data communication between nodes. Some distributed file systems such as GFS and Hadoop are already mature research topics. Achieving efficient and bandwidth-aware communication between nodes in a point-to-point manner requires Collective Communication Primitives. This is first introduced in high-performance computing systems and later brought into the field of deep learning. Modern deep learning frameworks use these primitives for the learning process. Because, it allows the gradient transfer between interconnected nodes to be completed in an optimal time. There are various variants in practical applications, such as recursive halving or doubling algorithms.

In distributed training, the configuration of computing and communication must be optimal to achieve effective horizontal scaling. Training is optimal if the communication steps are efficient and synchronized with the computations of the individual machines, i.e., computations across the cluster should end at roughly the same time. If the network is slow, the communication between nodes becomes a bottleneck. Gradient compression and mixed-precision training are both promising techniques that can increase the overall throughput of the network. A recent study has found that using a periodic learning rate reduces the number of nodes required to achieve network convergence by 10, making it a promising research direction for distributed training.

At present, the most common edge computing side feature fusion (Gai et al., 2019) has two methods, namely cascade and maximum pooling. Among them, the cascade is to splice and connect all the feature vectors. If a multi-view histogram is to represent cascading fusion. A column vector of MNK dimension can be obtained. Fusion can retain all the characteristic information of each view data. The function of maximum pooling is to calculate the maximum value of the feature vector and its one-to-one corresponding position in each view, as shown in equation (6):


(6) $$\begin{array}{r} \hat{{\text{r}}_{\text{i}}}\;=\;max_{\text{l}\leq \text{i}\leq \text{M}}r_{\text{ij}} \end{array}$$

M represents the total number of perspectives, and *r*_*ij*_ represents the j-th feature vector in the i-th perspective.

In the weighted feature fusion on the edge computing side, part of the image data is lost due to the compression of the model. This will affect the final output. Therefore, the pre-set dictionary size under each view is denoted as N_K_. The dictionary size of DDNN is MN_K_. M represents the total number of viewing angles. When the angle of view increases, the amount of DDNN calculations will also increase. In the MNK dictionaries, the data information carried by each view is relatively reduced, so the multi-view dictionaries are weighted, as shown in equations (7), (8) and (9):


(7) $$\begin{array}{r} {\text{c}}_{\text{i}}\;=\text{ concat}\left({\text{s}}_{\text{i}}^{\left(\text{l}\right)},\ldots ,{\text{s}}_{\text{i}}^{\left(\text{k}\right)},\ldots ,{\text{s}}_{\text{i}}^{\left(\text{M}\right)}\right) \end{array}$$

(8) $$\begin{array}{r} \text{scal}{\text{e}}_{\text{BoF}}\;=\text{ signmoid}\left({\text{c}}_{\text{i}}\right) \end{array}$$

(9) $$\begin{array}{r} \tilde{{\text{c}}_{\text{i}}}\;=\text{ scal}{\text{e}}_{\text{BoF}}\;\cdot \;{\text{c}}_{\text{i}} \end{array}$$

*c*_*i*_ represents the set of concatenating the histogram vectors of M views. $s_{i}^{\left(k\right)}$ represents the vertical quantity map of the k-th (1 ≤ k ≤ M) viewing angle. *scale*_*BoF*_ represents the importance score of each feature data.

Distributed training of neural networks can be achieved in two ways: data parallelization and model parallelization. The goal of data parallelization is to distribute the dataset equally across the nodes of the system. Each node has a copy of the neural network and its local weights. Each node processes a different subset of the dataset and updates its local set of weights. These local weights are shared across the cluster, resulting in a new global set of weights calculated through a cumulative algorithm. These global weights are in turn, distributed to all nodes, which then process the next batch of data based on that.

Model parallelization is to realize the distribution of training by dividing the model's architecture into different nodes. AlexNet is one of the earliest models to use model parallelization. The approach is to split the network across 2 GPUs so that the model fits in memory. Model parallelization can only be applied when the model architecture is too large to fit on a single machine, and some model parts can be parallelized. Model parallelization can be used in some models, such as object detection networks. The bounding box drawing and class prediction parts of this model are independent. In general, most networks can only be assigned to 2 GPUs. This limits the amount of scalability that can be achieved. Therefore, this paper mainly focuses on data parallelization and selecting the optimal training algorithms and communication primitives for different settings. The last two sections are the future research directions and conclusions, respectively.

A column is defined as a primitive integer column when its data type is larger than most required to store values. This means that its value occupies 4 bytes of storage. However, the current range of values in that column is 0 to 309. Therefore, recreating and reloading the table to use the encoding reduces the storage of each value in the column to 2 bytes.

This foreign key column encoding can be extended if the data values referenced by the other table are mostly 0 to 127. Before selecting, some data against the reference table needs to be queried to determine whether most values are in the 8-bit, 16-bit, or 32-bit range.

### Questionnaire on Student Learning Factors Adopting STEM Teaching Program

**Figure 5:**
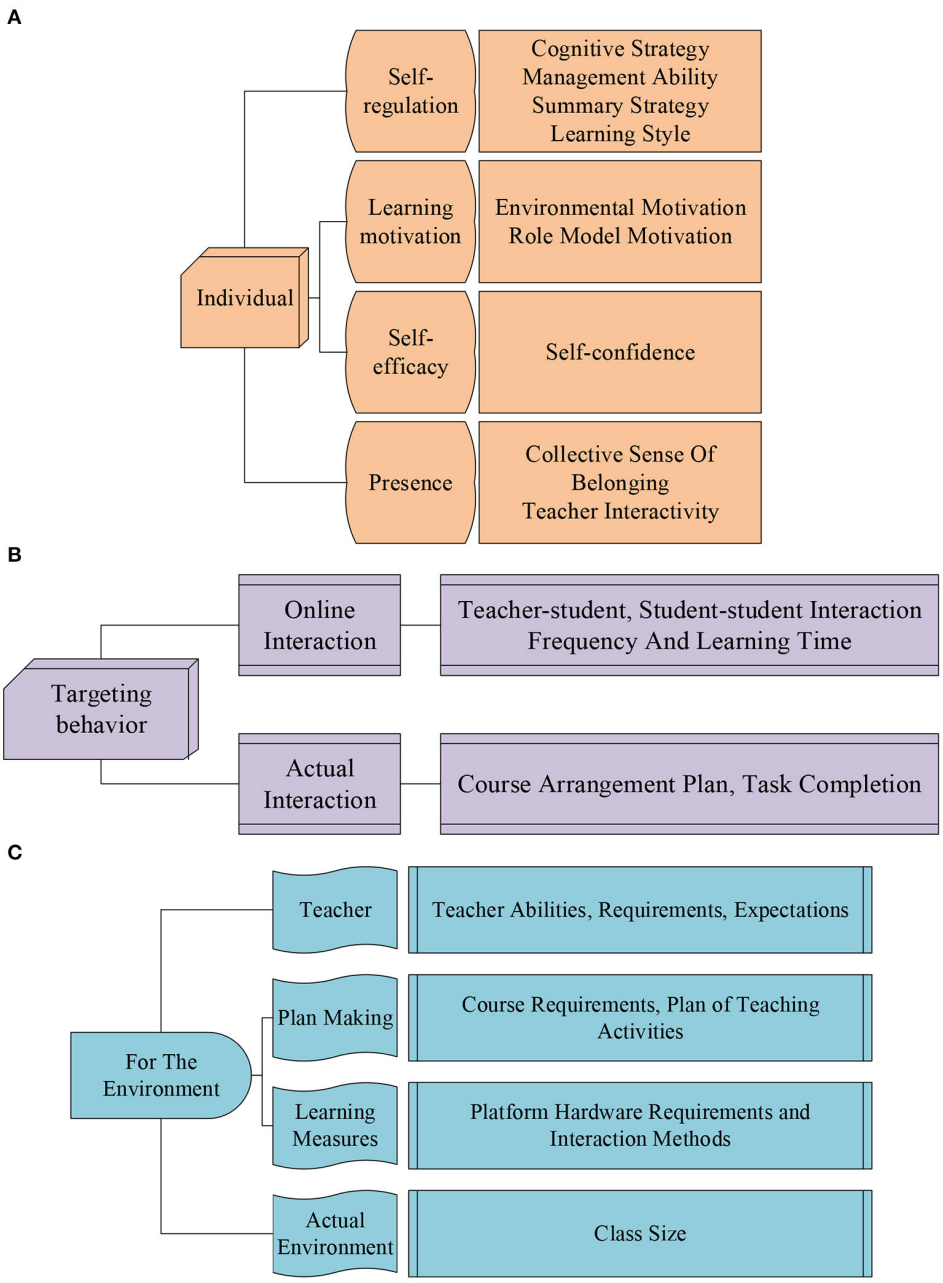
The impact dimension of students' deep learning. **(A)** Is the dimension for individual students, **(B)** is the dimension for student behavior, and **(C)** is the dimension for the environment.

The questionnaire is designed. In college courses, the questionnaire on the influencing factors of students' deep learning covers three dimensions, individual, behavior, and environment. The last part of the questionnaire is supplemented with an open question. These questions are supplemented by suggestions made by the students surveyed. Among them, the dimension description included in the questionnaire is shown in Figure 5. In order to ensure the validity of the questionnaire, repeated questions are avoided as much as possible.

**Figure 6:**
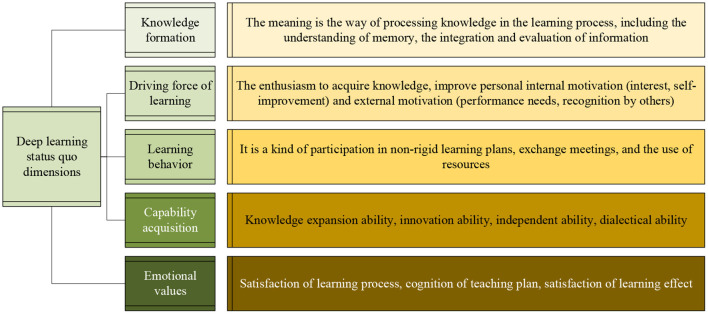
Dimensional description table of the status quo of deep learning.

The questionnaire contains questions about the formation of knowledge, the driving force of learning, the behavior of learning, the acquisition of abilities, and emotional values. The detailed description of each aspect is shown in Figure 6.

After referring to the relevant knowledge and suggestions, in the specific content of the designed questionnaire, there are a total of 5 options for each question. The evaluation criteria are: very disagree (1), disagree (2), general (3), more agree (4), very agree (5).

In a digital audio system, an A/D converter (often referred to as a sound card) is used to convert an analog signal into a digital signal (often, this process is called a recording). The sampling rate of the A/D converter is tens of thousands of times per second. Among them, each sample records the state of the sound wave at a certain moment. This thing is called a sample. When a series of samples is connected, it becomes a complete sound wave. The sampling rate determines the upper limit of the response frequency. When making digital copies of analog audio material, the minimum sampling rate of the material is 48 kHz. However, higher sample rates are achievable and may be more suitable for multiple types of audios. However, the higher audio sampling rate is beyond the range of human hearing. However, this high sampling rate and conversion technique improve audio quality within the range of human hearing. Unintentional or undesired artifacts in recordings are also part of the audio file, whether they are inherent in producing the file or later added to the original due to wear, mishandling, or poor preservation. High accuracy of preservation is guaranteed. For some specific signals and some types of noise, the sampling rate is preferably higher than 48 kHz. Although it is used as a reference standard, this is the upper limit. However, for most common audio material, the sample rate requested in the guidelines is sufficient. For original digital audio material, the sampling rate of the storage technology should be equal to the sampling rate of the original material.

After the questionnaire is designed, the details of the designed questionnaire are shown in Appendix Table A1. SPSS statistical software is used to analyze the questionnaire, and the results are shown in Table 2.

**Table 2:** Results of the questionnaire validity test.

**Sampling suitability measure**	**0.925**	
Sphericity test	Chi-square read	3,198.021
	Degree of freedom	405
	Significance	0.000

Among them, the number of sampling suitability is 0.925, which is greater than 0.9. The significance is 0.000. Therefore, judging by these two indicators, the validity of the questionnaire is good. The results can be used and adopted.

The questionnaire is aimed at college students in a certain place after using the teaching model. The learning effect is investigated, and 80 questionnaires are distributed. By controlling the survey subjects, they were all students who had received STEM teaching work. After 3 days, 73 questionnaires are recovered. Incomplete and defective, and invalid questionnaires are excluded. There are 69 valid questionnaires in total. The results are analyzed.

## Results

### STEM Teaching Program Model Training Results

**Figure 7:**
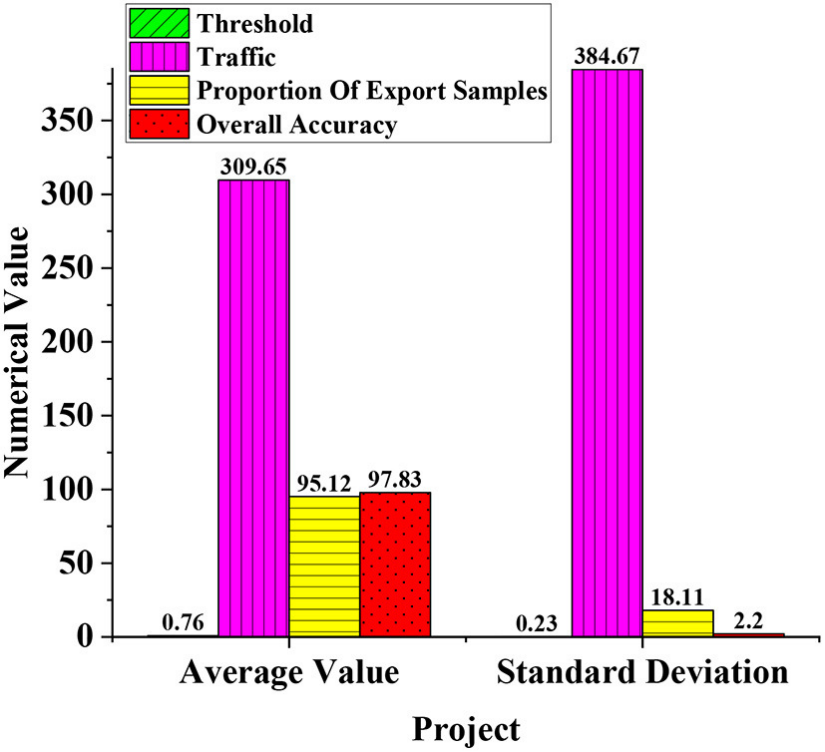
Parameters of STEM model training performance.

The average training method is used to verify the model's performance aiming at the proposed STEM teaching program model. Figure 7 is the model performance value obtained after using the average training method. The performance values are, respectively, the threshold, the amount of communication, the sample proportion of edge exits, and the overall accuracy. Among them, each analysis includes the mean and standard deviation. The results are analyzed after statistics.

In Figure 7, after the average training method, the average threshold is 0.76 and the standard deviation is 0.23. The average value of the communication volume is 309.65 bytes, and the standard deviation is 384.67 bytes. The mean value of the sample proportion of the marginal exit is 95.12, and the standard deviation is 18.11. The mean of the overall accuracy is 97.83%, and the standard deviation is 2.2%. Among them, the communication volume is 309.65 bytes, and the communication expectation is large, indicating that the communication volume performance is good. The sample ratio and overall accuracy of the edge exit are higher than 95%, indicating better overall performance. Therefore, the classification accuracy and communication performance of the STEM teaching program model after the average training method are at a relatively good level.

### The Statistical Results of the Questionnaire

**Figure 8:**
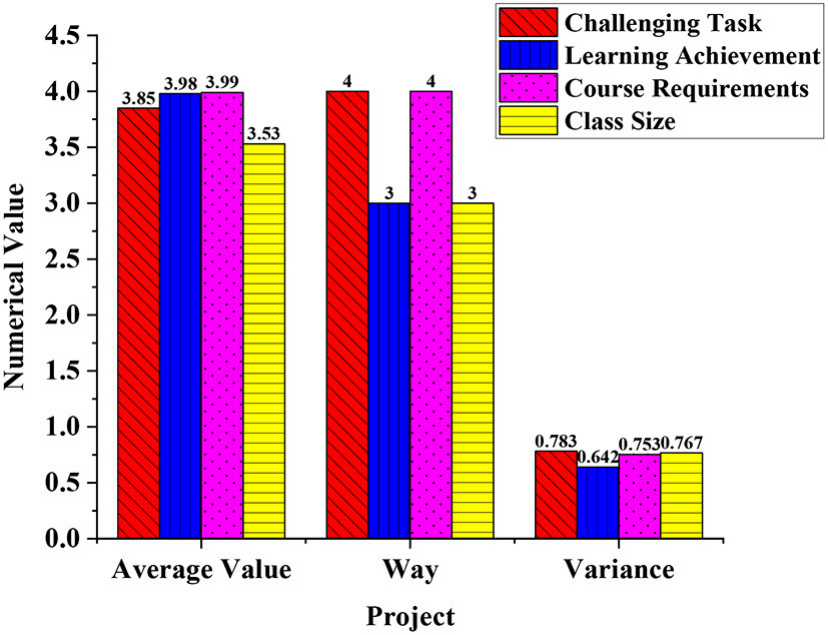
The impact of challenging tasks on deep learning.

Firstly, statistics of the impact of challenging problems on deep learning are shown in Figure 8.

The average value of the data reaches 3.85, the method is 4, and the variance is 0.783. The average value of the learning task for deep learning effect improvement reached 3.98, the model was 3, and the variance was 0.642. The average value of the course requirements for deep learning improvement is 3.99, the method is 4, and the variance is 0.753. The average improvement of class size for the deep learning effect is 3.53, the method is 3, and the variance is 0.767. More students support the emergence of challenging tasks to improve the effect of deep learning. And excluding the class size, the other three factors all have unity and influence each other. Due to the requirements of the course, challenging tasks appear. Completing the tasks will result in learning achievements and enhance students' recognition of the effects of deep learning.

**Figure 9:**
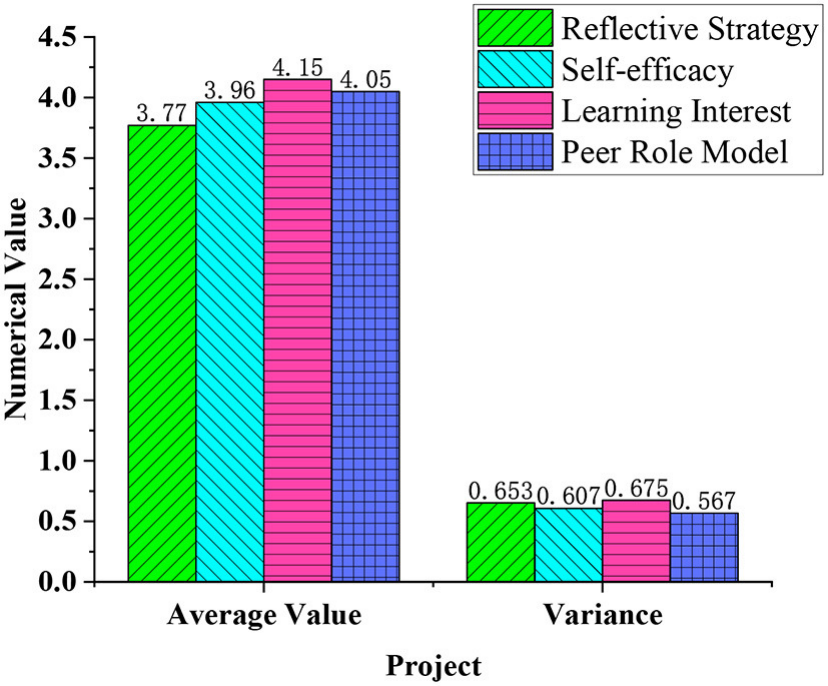
The degree of influence of factors such as learning motivation on deep learning.

Figure 9 shows the degree of influence of factors such as learning motivation on the effect of deep learning.

In Figure 9, the average value of the options for the reflection strategy's impact on deep learning is 3.77, and the variance is 0.653. The average effect of self-efficacy on deep learning is 3.96, and the variance is 0.607. The average effect of learning interest on deep learning is 4.15, and the variance is 0.675. Finally, the average impact of the role model students on deep learning is 4.05, and the variance is 0.567. The higher the confidence in learning and understanding courses, the better the effect of deep learning can be improved. This shows that the confidence state of the learner at the beginning of the learning process has a significant impact on the learning effect. Students with learning motivation and self-confidence can often understand the content of the course better and improve the effectiveness of their own deep learning. Therefore, in various innovative teaching programs, teachers need to focus on how to improve students' learning motivation and self-confidence in the course and provide a stable driving force for the improvement of deep learning effects.

## Conclusion

Deep learning has characteristics different from general learning, and its distinctive nature and characteristics are concentrated in attitude, span, and depth. Attitude refers to the fact that on the spiritual and psychological level, students are very engaged and focused on learning, have strong interest and enthusiasm, and participate deeply in the learning process. Span is a favorable way and method for students to take the initiative to break the barriers between disciplines and seek problem-solving from an interdisciplinary perspective and thinking. Depth is mainly reflected in inferences, analogy, and transfer applications in learning. Creatively solving problems in different situations is the inevitable requirement and concentrated expression of deep learning. In advocating the innovation and reform of educational methods, the issue of improving the effectiveness of deep learning in the teaching process of students has been raised. Innovative teaching plans are explored. DDNN under edge computing combined with deep learning related concepts, STEM teaching plan is proposed. The STEM teaching program model is established. Training methods are analyzed. The average training algorithm is used to validate the model. Related data performance is analyzed. After the STEM teaching program model has high accuracy and the amount of communication is obtained, the questionnaire is used to investigate the effect of deep learning for students who use the STEM teaching program in a university. Challenging tasks, learning motivation, and learning confidence impact knowledge comprehension and deep learning effects. Therefore, the proposed STEM teaching program can be applied in innovative teaching work. This can provide a certain reference concept for the work of relevant personnel. However, the training method for the performance of the model is not fully used here, and only the average algorithm is used as a training method. In the future, more training methods will be used to verify the model to illustrate its good performance further.

## Data Availability Statement

The raw data supporting the conclusions of this article will be made available by the authors, without undue reservation.

## Ethics Statement

The studies involving human participants were reviewed and approved by Physical Education College of Zhengzhou University Ethics Committee. The patients/participants provided their written informed consent to participate in this study. Written informed consent was obtained from the individual(s) for the publication of any potentially identifiable images or data included in this article.

## Author Contributions

All authors listed have made a substantial, direct, and intellectual contribution to the work and approved it for publication.

## Funding

This work was supported by Training Program for Young Backbone Teachers in Higher Education Institutions in Henan Province in 2020 (No. 2020GGJS294).

## Conflict of Interest

The authors declare that the research was conducted in the absence of any commercial or financial relationships that could be construed as a potential conflict of interest.

## Publisher's Note

All claims expressed in this article are solely those of the authors and do not necessarily represent those of their affiliated organizations, or those of the publisher, the editors and the reviewers. Any product that may be evaluated in this article, or claim that may be made by its manufacturer, is not guaranteed or endorsed by the publisher.

## Appendix

**Table A1:** Questionnaire on factors affecting deep learning.

**Number**	**Question content**	**Evaluation criteria**				
1	New understanding and memory of new knowledge during learning	1	2	3	4	5
2	Ability to compare new knowledge with existing knowledge during learning	1	2	3	4	5
3	New knowledge is evaluated during learning	1	2	3	4	5
4	Learning motivation in the process of learning	1	2	3	4	5
5	Interested in the course content	1	2	3	4	5
6	Study because of course requirements	1	2	3	4	5
7	In order to obtain the recognition of others to learn	1	2	3	4	5
8	In order to improve self-ability to learn	1	2	3	4	5
9	Participated in non-rigid requirements activities, the pursuit of practical results	1	2	3	4	5
10	Actively interacting with others to improve learning effect	1	2	3	4	5
11	Can apply new knowledge in the classroom	1	2	3	4	5
12	Raise one's ability through new knowledge	1	2	3	4	5
13	New knowledge enhances my ability to think	1	2	3	4	5
14	New knowledge helped me gain more positive values	1	2	3	4	5
15	I am very satisfied with deep learning	1	2	3	4	5
16	The course plan will affect the effect of deep learning	1	2	3	4	5
17	More summary and reflection can improve deep learning effect	1	2	3	4	5
18	Interest in the course can improve deep learning	1	2	3	4	5
19	The higher the performance requirements, the better the deep learning effect	1	2 3	4	5
20	The higher the self-identity after learning, the better the deep learning effect	1	2	3	4	5
21	Learning styles and styles affect deep learning	1	2	3	4	5
22	The more active the learning group is, the better the deep learning effect is	1	2 3	4	5
23	High frequency of interaction with teachers can improve deep learning effect	1	2	3	4	5
24	The more offline course arrangement, the deeper learning effect	1	2	3	4	5
25	The longer the online learning time, the better the deep learning effect	1	2	3	4	5
26	The higher the quality of learning tasks, the better the deep learning effect	1	2	3	4	5
27	Learning motivation is stimulated by learning tasks, and deep learning is effective	1	2	3	4	5
28	The higher the confidence in performance, the better the deep learning effect	1	2	3	4	5
29	The more challenging tasks, the more you can improve deep learning	1	2	3	4	5
30	Other factors affecting deep learning:					
